# Transforaminal lumbar interbody fusion with a tantalum cage: lumbar lordosis redistribution and sacral slope restoration with a modified posterior technique

**DOI:** 10.1186/s10195-023-00741-3

**Published:** 2023-12-13

**Authors:** Marcello Ferraro, Francesco Puglia, Andrea Della Valle, Vincenzo Cerbone, Alfonso Cicatelli, Donata Rita Peroni, Davide Cecconi, Bernardo Misaggi, Giovanni Andrea La Maida

**Affiliations:** 1 Spine Surgery Department, Orthopaedic Institute Gaetano Pini, Via Gaetano Pini, 1, 20121 Milan, Italy; 2 University of Milan, Milan, Italy

**Keywords:** Tantalum, Cage, Lumbar lordosis, TLIF, Interbody fusion

## Abstract

**Background:**

Transforaminal lumbar interbody fusion (TLIF), a commonly used procedure in spine surgery, has the advantage of a lower incidence of nerve lesions compared to the posterior lumbar interbody fusion (PLIF) technique. The intersomatic arthrodesis has always been carried out with a single tantalum cage normally used for PLIF. Tantalum is a metal that is particularly used in orthopedic surgery. It has a modulus of elasticity similar to marrow and leads to high primary stability of the implant.

**Materials and methods:**

Our study was a retrospective monocentric observational study evaluating clinical and radiological outcomes of tantalum cages in a modified TLIF technique with posterior instrumentation and autologous and/or homologous posterolateral bone grafting. The aim of the study was to evaluate clinical outcomes and the increase in or redistribution of lumbar lordosis. The intersomatic arthrodesis was always carried out with a single tantalum cage normally used for PLIF to reduce the neurological risk. We retrospectively studied 105 patients who were treated with a modified unilateral TLIF approach by two surgeons between 2013 and 2018. We evaluated the Oswestry Disability Index (ODI), Visual Analogue Scale (VAS) for back pain, global lumbar lordosis, lordosis of L4–sacrum, segmental lordosis of functional motion units that underwent arthrodesis, pelvic tilt, pelvic incidence, and the sacral slope in 77 patients. All patients were suffering from grade III or IV Pfirrmann, instability, or foraminal post-laminectomy stenosis and/or grade I–II degenerative spondylolisthesis or low-grade isthmic spondylolisthesis. They had no significant sagittal imbalance, with a sagittal vertical axis (SVA) of < 5 mm. The average follow-up duration was 30 months.

**Results:**

We achieved excellent clinical results, with only four cases of failure (5.2%). Moreover, we noticed a statistically significant redistribution of lumbar lordosis, with an average percentage increase in L4–S1 lordosis equal to 19.9% (*P* < 0.001), an average increase in the L4–S1/Lumbar lordosis (LL) ratio from 0.53 to 0.63 (*P* < 0.001), and a mean percentage increase in sacral slope equal to 7.6% (*P* < 0.001).

**Conclusion:**

Thanks to the properties of tantalum, our modified single-portal TLIF technique is a valid surgical solution to obtain a solid arthrodesis and restore the correct lumbar lordosis distribution while reducing neurological complications and the number of failures.

*Level of evidence*: 4

*Trial registration statement*: retrospective observational study, no trial registration.

## Introduction

Lumbar interbody fusion with posterior instrumentation is a universally accepted spinal surgery technique that is considered at the end of appropriate conservative treatments. Intersomatic arthrodesis allows a solid and effective intervertebral fusion to be obtained, together with indirect decompression of nervous structures [1, 2].

The surgical landscape for treating various spinal pathologies has evolved over the years, with a myriad of techniques now on offer, including posterior lumbar interbody fusion (PLIF), transforaminal lumbar interbody fusion (TLIF), oblique lumbar interbody fusion/anterior to psoas (OLIF/ATP), lateral lumbar interbody fusion (LLIF), and anterior lumbar interbody fusion (ALIF) [3, 4]. This study focuses on a modified monolateral TLIF technique that uses tantalum cages with the aim of reconciling the benefits of different approaches while mitigating their drawbacks.

Our technique emerges from the clinical need to improve outcomes and lower complications. Although ALIF is effective and reduces post-operative hospitalization [6–8], it presents a higher risk for vascular and visceral complications [5]. While PLIF facilitates direct neural decompression [9], it is associated with an increased risk of nerve root damage and dural tears [10]. Our choice of the modified monolateral TLIF aims to combine the clinical advantages of both these techniques, in alignment with existing literature [11].

Tantalum offers distinct advantages. Its modulus of elasticity closely mimics that of bone marrow, aiding in stress distribution and reducing the likelihood of implant loosening [12–14]. It also has an increased coefficient of friction when compared to autografts or allografts, thus enhancing implant stability [13, 15–19]. Given these properties, tantalum-based cages have shown promising outcomes in prior studies involving both cervical and lumbar fusions [20–24].

However, it is important to acknowledge the limitations of our approach. While tantalum demonstrates lower bacterial adhesion with* Staphylococcus aureus*, it does not inhibit biofilm formation or demonstrate broad antibacterial action [25–37]. Additionally, the TLIF technique inherently carries a risk of nerve lesions, albeit a lower risk than that from PLIF [11].

Our modified monolateral TLIF approach with tantalum cages seeks to blend the nerve-preserving features of TLIF with the muscle-sparing properties of ALIF. The literature on TLIF reports lower pain indices based on the Oswestry Disability Index (ODI) score compared to ALIF and PLIF, though no differences were noted in the VAS score [11]. Our work expands upon these findings by incorporating tantalum as an optimal material for cage construction, given its aforementioned benefits and limitations.

In summary, this retrospective, monocentric, observational study aims not only to assess clinical outcomes but also to examine changes in lumbar lordosis following intersomatic arthrodesis. It represents an amalgamation of current best practices and innovative materials, offering a nuanced solution in a complex surgical landscape.

## Materials and methods

### Patient selection

Our retrospective study analyzed 105 patients who underwent posterior instrumented arthrodesis with pedicle screws and intersomatic arthrodesis with a modified unilateral TLIF approach using a tantalum cage and autologous and/or homologous postero-lateral bone grafting between 2013 and 2018. All the autologous bone was harvested from the decompression area.

Patients treated with this surgical technique had been suffering from lumbar back pain resistant to conservative treatment for more than 6 months with or without leg pain. Such patients were also afflicted with grade III or IV discopathy according to Pfirrmann (with or without peripheral symptoms), instability, or foraminal post-laminectomy stenosis and/or grade I–II degenerative spondylolisthesis or low-grade isthmic spondylolisthesis. All patients examined had a sagittal vertical axis (SVA) of < 5 mm.

In each case, an accurate pre-operative surgical plan with standard routine radiographs was made: a lumbosacral spine X-ray in two views and a dynamic X-ray and full-length spine X-ray in a lateral view only, with evaluations of global lumbar lordosis, lordosis of L4–sacrum, segmental lordosis of functional motion units that underwent arthrodesis, pelvic tilt, pelvic incidence, and the sacral slope. In addition, a careful clinical evaluation was performed using the VAS back and ODI scales [28].

### Surgical technique

**Fig. 1:**
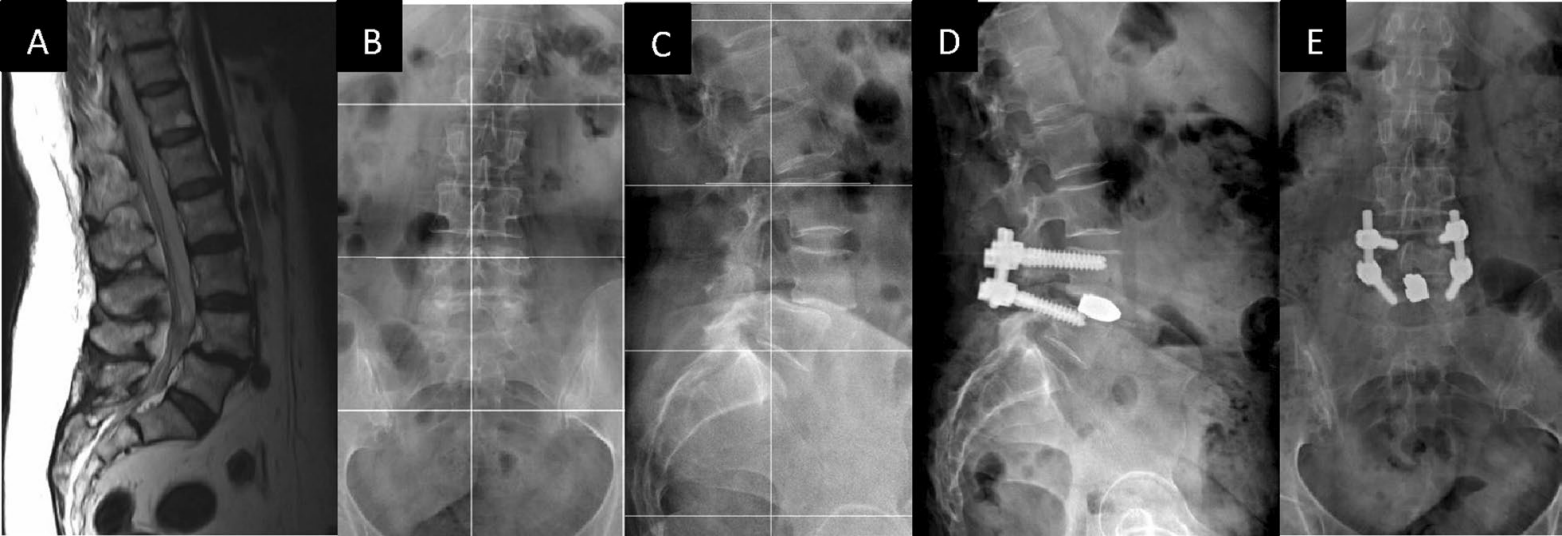
**A** Pre-op MRI with L4–L5 discopathy and spondylolisthesis. Lumbosacral spine X-rays: **B** pre-op AP view, **C** pre-op lateral view (L4–L5 lordosis 4°, L4–S1 lordosis 30°), **D** post-op lateral view (L4–L5 lordosis 18°, L4–S1 lordosis 38°), **E** post-op AP view

After general anesthesia, all patients always underwent surgery performed by the same two surgeons with the same surgical technique: Bone exposure of the region and placement of bilateral pedicle screws with a free-hand technique followed by ampliscopic control. The intersomatic arthrodesis was always carried out by "widened" monoportal TLIF access, enlarging the traditional technique of foraminotomy towards the lamina to permit the initial oblique insertion of a single tantalum cage normally used for PLIF (but usually with a smaller size) to reduce the neurological risks. The positioning of the cage was preceded by an accurate discectomy and the preparation of the vertebral end plates using dedicated curettes, and an attempt was made to place the final cage in the most central and anterior position possible. All cages used were 9 mm wide and 22 mm long, the smallest available size of TM Ardis (Zimmer Spine); a width of 9 mm was chosen to reduce the volume, and a length of 22 mm was chosen to maximize the wedge effect with the posterior cantilever in order to reduce the risk of neurological compressions and optimize intersegmental lordosis (Fig. 1). The only variation was due to the different height of the chosen device in relation to the space between the two vertebral end plates. With the aim of obtaining a solid intervertebral fusion, we placed autologous bone chips from the decompression area on the side of every cage between the vertebral bodies and then added autologous and/or homologous posterolateral bone grafting bilaterally.

### Follow-up

All patients wore a semi-rigid lumbosacral corset in orthostasis and/or the seated position for the following 3 post-operative months.

Post-operative checks were performed using a lumbosacral spine X-ray in two views and full-length spine X-rays in lateral view only, evaluating the same spine parameters as above, and a clinical evaluation was performed using VAS back and the Oswestry Disability Index (ODI) [31].

Seventy-seven of the 105 patients with a mean age of 53.8 years (range 21–72 years) were recruited into the study for a minimum 1 year of follow-up (Table 1). Patients underwent their first clinical evaluation and X-rays 3 months after the surgical procedure, and they were followed every 6 months by performing a new clinical and radiological check. At 1 year of follow-up, all patients underwent a CT scan of the affected segments to assess the fusion rate.

**Table 1:** Baseline characteristics

	Total modified TLIF	TLIF L4–L5	TLIF L5–S1	TLIF L4–L5 + L5–S1
No.	77	48	15	14
Age (years)	53.9	58.1 ± 11.1	42.0 ± 14.0	52.3 ± 12.2
Sex	38M/39F	24M/24F	6M/9F	8M/6F

The average follow-up duration in the study was 30 months (range 24–58 months).

### Statistical analysis

The statistical analysis was performed using SPSS 19.0. The following descriptive variables were obtained: average, range, standard deviation, and distribution frequency. Comparisons between pre- and post-operative values were made using the two-tailed *T* test. Significance was attributed when *P* < 0.05.

## Results

### Clinical

We achieved excellent clinical results, with an average reduction in the VAS back from 8 to 2 points (*P* < 0.05). On the ODI scale, we found at the follow-up that 66 patients (85.7%) had minimal disability, 7 patients (9.1%) had moderate disability, 2 patients had severe disability (2.6%), and 2 patients had the greatest disability (2.6%). The mean ODI score was 42.08 ± 11.90 before the surgery and 11.12 ± 14.02 at the follow-up, indicating a statistically significant reduction (*P* < 0.01).

### Radiological

**Fig. 2:**
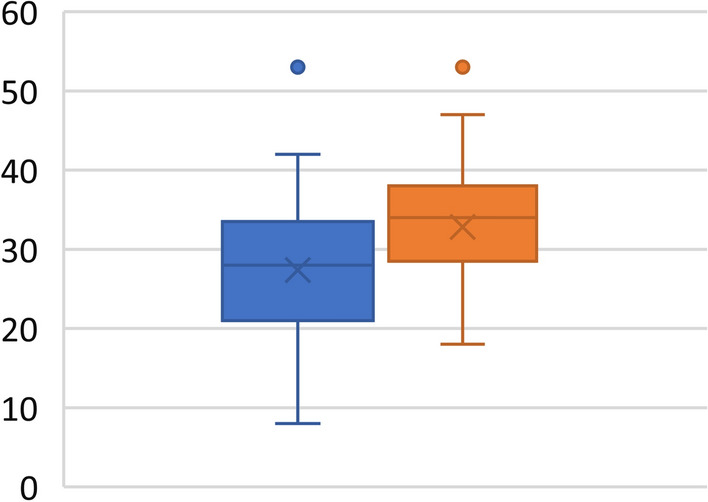
Increase in L4–S1 lordosis in the study

Our radiological results were based on the desire to know if a single-portal TLIF cage can restore lumbosacral lordosis. In all 77 patients with an average age of 53.8 years (± 13.3), we noticed an average increase in L4–S1 lordosis of 5.4° (*P* < 0.001), equal to a mean percentage increase of 19.9%, with the L4–S1/LL lordosis ratio increasing on average from 0.53 to 0.63 (*P* < 0.001) (Fig. 2). This corresponds to a statistically significant redistribution of lumbar lordosis without a significant increase in global lumbar lordosis but with an average increase in the sacral slope of 2.7°, equal to a mean percentage increase of 7.6% (*P* < 0.001).

**Fig. 3:**
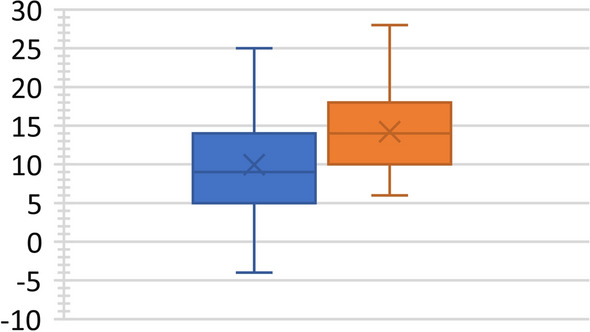
Segmental lordosis increase in patients with a cage at L4–L5

**Fig. 4:**
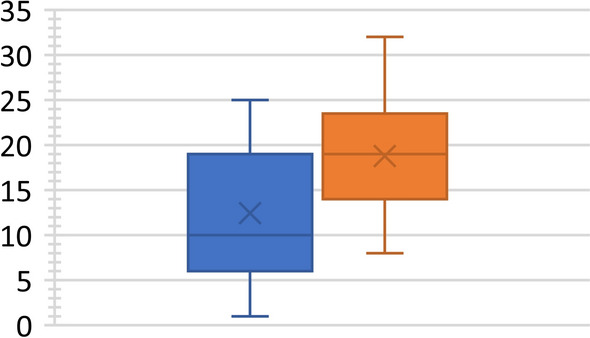
Segmental lordosis increase in patients with a cage at L5–S1

The sample was divided into two groups: 62 patients with a cage at L4–L5 (31 men and 31 women with an average age of 56.7 ± 11.5 years) and 29 patients with a cage at L5–S1 (14 men and 15 women with an average age of 46.9 ± 13.1 years); note that 14 patients with intersomatic arthrodesis of the two levels were included in both groups. The data showed that the value of LL was practically unchanged compared to the pre-operative value. However, there were statistically significant mean increases in segmental lordosis of 4.3° in the patients with an L4–L5 cage, equal to a mean percentage increase of 42.8% (*P* < 0.001) (Fig. 3), and of 4.7° in patients with an L5–S1 cage, equal to a mean percentage increase of 38.1% (*P* < 0.001) (Fig. 4).

Then, we continued the statistical analysis by detecting the grade L4–S1 lordosis, this time dividing the sample into three groups: 48 patients with intersomatic arthrodesis at L4–L5 (composed of 24 men and 24 women with an average age of 58.0 ± 11.1 years; Table 2), 15 patients with intersomatic arthrodesis at L5–S1 (composed of 6 men and 9 women with a mean age of 42.0 ± 14.0 years; Table 3), and 14 patients with intersomatic arthrodesis at both L4–L5 and L5–S1 (composed of 8 men and 6 women with a mean age of 52.2 ± 12.2 years; Table 4). The data showed statistically significant average increases in L4–S1 lordosis in patients with L4–L5 cage implantation (increase of 3.8°, equal to a mean percentage increase of 13.7%; *P* < 0.001), in patients with a L5–S1 cage (increase of 4.7°, equal to a mean percentage increase of 15.9%; *P* < 0.001), and in patients with L4–L5 and L5–S1 cages (increase of 11.7°, equal to a mean percentage increase of 52.9%; *P* < 0.001). In particular, in the last group with two cages at adjacent levels, we also found an average increase in global lordosis of 9.1°, equal to a mean percentage increase of 21.6% (*P* < 0.001).

**Table 2:** Patients with an L4–L5 intersomatic cage. Pelvic incidence (PI), Sacral Slope (SS), Pelvic Tilt (PT)

Cage at L4–L5	Pre-operative	Follow-up	*P *value
PI	58.8 ± 9.8	59.3 ± 9.5	–
SS	35.6 ± 10.9	37.5 ± 10.6	< 0.05
PT	23.2 ± 8.8	21.8 ± 8.5	0.05
LL	51.9 ± 11.9	52.4 ± 11.9	0.33
L4–L5	10.4 ± 5.3	14.4 ± 4.5	< 0.0001
L4–S1	28.1 ± 8.7	32.0 ± 7.0	< 0.0001
L4–S1/LL	0.54 ± 0.14	0.62 ± 0.14	< 0.0001

**Table 3:** Patients with an L5–S1 intersomatic cage

Cage at L5–S1	Pre-operative	Follow-up	*P *value
PI	60.5 ± 8.1	61.3 ± 9.0	–
SS	39.0 ± 8.4	41.9 ± 9.6	< 0.05
PT	21.5 ± 7.8	19.4 ± 8.5	0.09
LL	59.5 ± 6.5	58.9 ± 10.3	0.40
L5–S1	14.4 ± 7.4	19.5 ± 6.4	< 0.0001
L4–S1	29.7 ± 5.9	34.4 ± 6.0	< 0.001
L4–S1/LL	0.50 ± 0.11	0.60 ± 0.16	< 0.05

**Table 4:** Patients with L4–L5 and L5–S1 intersomatic cagess

Cages at L4–S1	Pre-operative	Follow-up	*P* value
PI	54.3 ± 11	55.9 ± 9.2	–
SS	32.3 ± 12.3	37.6 ± 10.5	< 0.05
PT	22.1 ± 9.7	18.3 ± 7.9	0.05
LL	42.3 ± 14.1	51.5 ± 11.0	< 0.001
L4–L5	8.4 ± 7.3	13.3 ± 5.8	< 0.05
L5–S1	10.3 ± 6.5	18.1 ± 6.6	< 0.0001
L4–S1	22.1 ± 10.0	33.8 ± 6.6	< 0.0001
L4–S1/LL	0.53 ± 0.16	0.67 ± 0.09	< 0.001

Upon analyzing CT scans obtained at the 1-year follow-up, we observed complete vertebral fusion of the involved spinal segments in 85 of 91, with a fusion rate of 93.4%.

### Complications

The following complications were reported for our series: three dural tears without neurological damage (3.9%), of which only one needed surgical revision (1.3%); one dural tear with hyposthenia of the peroneal muscles contralateral to the TLIF portal, with total recovery at 12 months (1.3%); ten patients with transient sciatic nerve pain (13.0%) with an average persistence of 5 post-op days, which caused an increase in the duration of hospitalization; four cases of asymptomatic subsidence (5.2%) caused by technical errors in the positioning of the cage related to excessive cruentation of the vertebral end-plate (this was demonstrated by the fact that we did not find further depression in the first and subsequent post-operative X-rays compared to the intraoperative radiographs); one sagittal imbalance with SVA > 5 mm caused by incorrect preoperative planning (1.3%); one loosening of the posterolateral instrumentation with concomitant loosening of the cage, causing mechanical failure of the implant (1.3%); one case of extruded hernia at an adjacent level 2 years after surgery (1.3%); and one loosening of the implanted cage after 6 months with subsequent surgical revision (1.3%).

Therefore, four cases of failure that required surgical revision were found at follow-up (a rate of 5.2%), of which three failures were due to mechanical issues (3.9%) and one was due to dural injury during the surgical procedure (1.3%).

## Discussion

**Fig. 5:**
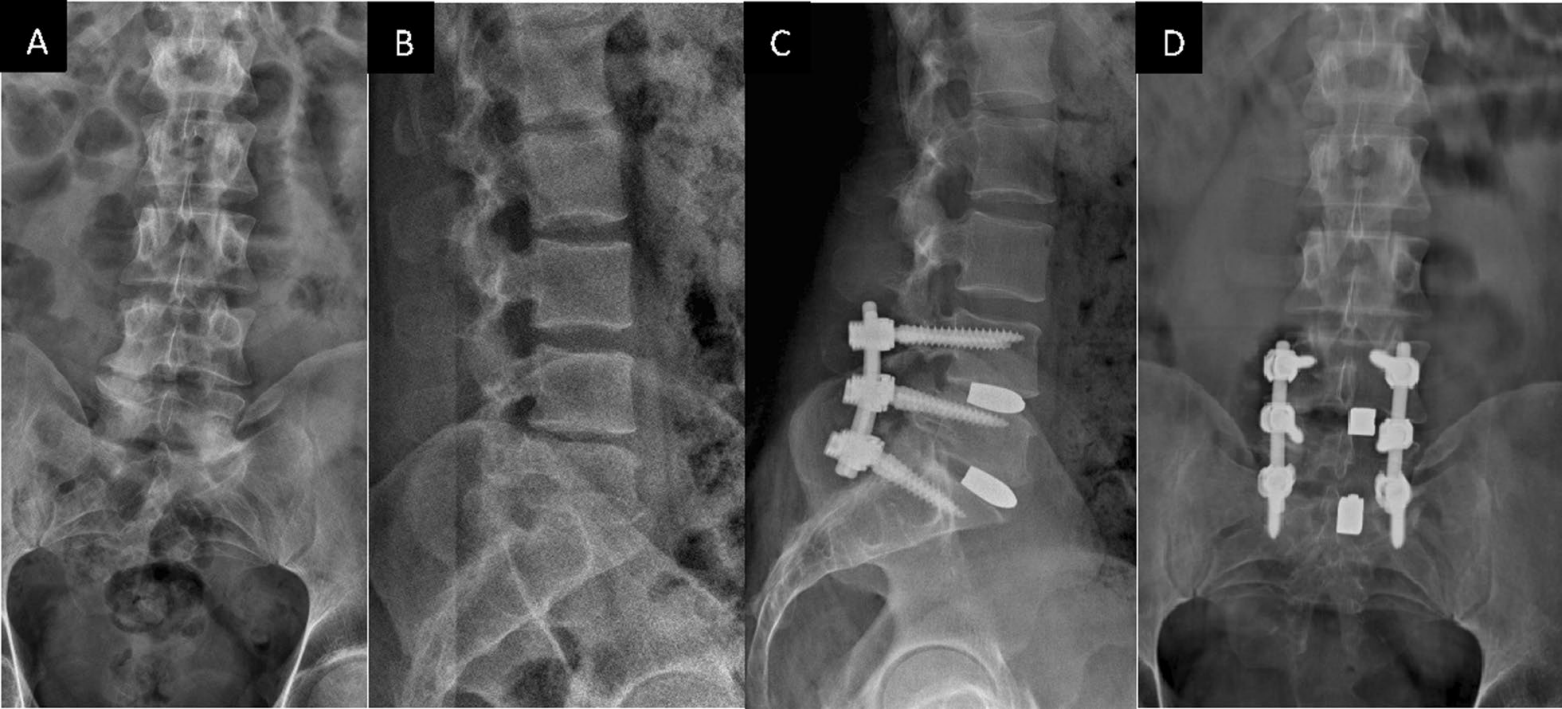
Lumbosacral spine X-ray: **A** pre-op AP view, **B** pre-op lateral view (PT 18°, SS 16°, L4–S1 lordosis 13°, global lumbar lordosis 24°), **C** post-op lateral view (PT 7°, SS 27°, L4–S1 lordosis 29°, global lumbar lordosis 41°), **D** post-op AP view

Intersomatic arthrodesis remains a milestone in spine surgery, as it is a fundamental aid to the post-operative biomechanics of a spine thanks to the support given to the anterior column. This allows for better load transfer and minimizes the risk of mobilization/non-osteointegration of the posterior instrumentation to promote solid callus formation. The positioning of cages with the single-portal TLIF technique on one or more intervertebral levels allows for a lower risk of dural and radicular lesions compared to the PLIF technique [29], as the approach to the disc is more distant/lateral to the spinal cord. Based on our experience, we decided to make the entrance point slightly more oblique, modifying the original surgical technique with a partial laminotomy and flavectomy in order to introduce a single smaller PLIF cage at the intersomatic position, taking into consideration the high friction coefficient of the material [20] (and further reducing the risk of neurological complications). We placed the cages in the most central and anterior position possible, which requires an appropriate learning curve (Fig. 5). The aim of using a central position is to obtain valid support and reduce vectorial forces on the anterior spine. The aim of adopting an anterior position is to achieve a greater wedge effect on the functional motion unit, which, together with the compression through posterior instrumentation, gives lordosis, as shown by TLIF cantilevers by Kida et al. [30]. However, in our case report, a coronally asymmetric cage position was not directly related to biomechanical and/or symptomatic complications of the patients in the study. This is explained by the properties of tantalum, including its high osteoconductive capacity [31, 32], which, together with its high biocompatibility and resistance [13], allows excellent osteointegration [14, 15] and a reduction in the stress-shielding effect, minimizing the risk of mobilization [33–35]. In addition, the trabecular morphology of the cage ensures bone ingrowth, promoting total adhesion between the vertebral end-plates involved in the arthrodesis: in fact, the proliferation of osteoblasts in vitro was demonstrated to be 6 times greater than with titanium fiber mesh and 12–16 times greater than with culture plastic [36].

This technique also allowed us to drastically reduce the surgical time compared with conventional TLIF. There was also a reduction in cost compared with TLIF procedures, as also demonstrated in a recent comparative analysis [37].

Sinclair et al. have shown that tantalum has a higher stability compared to PEEK (polyether ether ketone) in animal models due to a higher volume of bone ingrowth [13]. Recent studies on an animal model have also shown greater bone growth in porous titanium cages printed in 3D compared to PEEK [38]. However, there are no studies comparing this new material with porous tantalum.

In a series of 50 patients who underwent the PLIF procedure with a tantalum cage, Mallory et al. showed that complete fusion was present in 96% of patients at 1 year and in 100% of patients at 2 years of follow-up [39].

It should also be reiterated that tantalum cages lead to fewer artifacts in the T1 and T2 spin echo and gradient echo sequences in MRI, allowing better visualization of neurological structures [40], although it causes more CT artifacts.

In a recent review [41], a fusion rate of 93.1% was demonstrated for the MI-TLIF technique, while the complication rate was 9.6%.

We maintain that the accurate patient selection and properties of the aforementioned material contributed significantly to the low failure rate (5.2%) and the fusion rate at 1 year of follow-up (93.4%) achieved in this work, which are similar to those in other studies with tantalum cages [40, 41]. In our view, the combination of the surgical technique and the increase in and/or redistribution of LL and segmental lumbar lordosis following intersomatic arthrodesis obtained with this type of device should not be underestimated.

Upon measuring the pelvic spine angles with LL of all the patients who underwent a graphic X-ray check at a mean follow-up of 30 months, we found an average lordosis increase in the single segment of 42.8% (*P* < 0.001) in patients with an L4–L5 cage and 38.1% (*P* < 0.001) in patients with an L5–S1 cage.

But, in our opinion, the most important information is the redistribution of global lordosis. There was a mean increase in L4–sacrum lordosis of 5.4° (19.9%; the increase was directly proportional to the number of TLIFs performed), with an average increase in sacral slope of 2.7° (*P* < 0.001), even without a clear increase in global lordosis. For global lordosis, and especially in revision surgery with a serious or important sagittal imbalance, the value of ALIF is self-evident thanks to its direct action against the anterior longitudinal ligament and the use of hyperlordotic cages. However, it is not free from a high percentage of vascular and visceral complications and a high incidence of cage migration [3, 29].

We believe that the technique used in our study should be compared against the use of lordotic cages and expandable cages. In our experience, the use of a lordotic cage makes it difficult to position the intersomatic device in marked discopathy (Pfirmann IV), especially if it is associated with spondylolisthesis, due to the larger anterior portion of the cage. Moreover, Lee et al. compared cages with different degrees of lordosis and showed that the same clinical and radiographic results were obtained with a short-level PLIF [42].

Expandable cages, on the other hand, are not only unrelated to better correction of global lumbar lordosis, as demonstrated by Alvi et al. in their meta-analysis, but they have also a smaller fusion area (due to the expansion mechanism itself) and a higher cost [43].

There are also some limitations of this study. Firstly, its small patient sample limits the extent to which the findings can be generalized. Secondly, the study lacks a comparative analysis with other commonly used materials like PEEK or porous titanium, which leads to an incomplete understanding of tantalum's efficacy. Additionally, the focus on specific medical conditions may introduce a bias into the study. This could potentially lead to results that overly favor the positive clinical implications of using tantalum in TLIF procedures.

## Conclusion

Our modified single-portal TLIF technique with a tantalum cage is a valid surgical solution in patients with disc pathologies with or without symptoms of radicular/foraminal stenosis and/or non-severe hypolordosis. It gives excellent clinical and radiographic results and is a good compromise between solid arthrodesis, restoration of the correct lumbar lordosis distribution, and both direct and indirect decompression of the neurological structures in a single surgical session.

The contribution of tantalum to obtaining excellent clinical outcomes is remarkable. It allows a significant increase in segmental lordosis with a better redistribution of LL and excellent primary stability, significantly reducing the number of failures and reoperations.

## Data Availability

All data generated or analyzed during this study are included in this published article and its additional files.
